# Hydrogels Modulating the Microbiome: Therapies for Tissue Regeneration with Infection Control

**DOI:** 10.3390/gels11080584

**Published:** 2025-07-29

**Authors:** Germán Reynaldo Jiménez-Gastelum, Carlos Esteban Villegas-Mercado, Juan Luis Cota-Quintero, Silvia Ivonne Arzola-Rodríguez, Rosalío Ramos-Payán, Mercedes Bermúdez

**Affiliations:** 1 Faculty of Biology, Autonomous University of Sinaloa, Culiacan PC 80013, Mexico; germanjimenez@uas.edu.mx (G.R.J.-G.); luis@uas.edu.mx (J.L.C.-Q.); 2 Faculty of Odontology, Autonomous University of Chihuahua, Chihuahua PC 31000, Mexico; cmercado@uach.mx (C.E.V.-M.); sarzola@uach.mx (S.I.A.-R.); 3 Faculty of Biological and Chemical Sciences, Autonomous University of Sinaloa, Culiacan PC 80013, Mexico; rosaliorp@uas.edu.mx

**Keywords:** Hydrogels, Hydrogel-based scaffolds, microbiome, microbiome modulation, tissue regeneration, infection control

## Abstract

This review explores the emerging role of functionalized hydrogels in modulating the microbiome for therapeutic applications in tissue regeneration and infection control. The skin and gut microbiomes play crucial roles in maintaining tissue homeostasis, regulating immune responses, and influencing the healing process. Disruptions in microbial balance—such as those observed in chronic wounds, autoimmune conditions, or post-surgical environments—can impair regeneration and increase susceptibility to infection. Hydrogels, due to their tunable physical and chemical properties, serve as versatile platforms for delivering probiotics, prebiotics, antimicrobials, and immune-modulatory agents. The encapsulation of beneficial bacteria, such as *Lactobacillus plantarum* or *Prevotella histicola*, within hydrogels could enhance bacterial viability, targeted delivery, and immune tolerance. Additionally, hydrogels functionalized with silver nanoparticles, nitric oxide donors, and bacteriocins have demonstrated effective biofilm disruption and pathogen clearance. These systems also promote favorable immune responses, such as M2 macrophage polarization and the induction of regulatory T cells, which are essential for tissue repair. Innovative approaches, including 3D bioprinting, self-healing materials, and photothermal-responsive hydrogels, expand the clinical versatility of these systems.

## 1. Introduction

The skin is the largest sensory organ in the body, acting as the primary physical-chemical barrier between the environment and the interior of an individual’s body [1]. Histologically, the skin (or common integument) is made up of two layers: the epidermis and the dermis; the epidermis is the outermost layer made up of stratified keratinized squamous epithelium in four different layers. Meanwhile, the dermis is a layer of dense tissue, divided into two main layers: the papillary layer and the reticular layer. Beneath the dermis lies the hypodermis, a layer of loose connective tissue containing adipose cells [1]. Among the skin’s primary functions is protection as a barrier through physiological responses to mechanical abrasion, as well as immune responses [2,3,4]. Additionally, the skin represents a suitable surface for colonizing microorganisms, which participate symbiotically with the individual’s immunological mechanisms in protection [2,3,4].

During birth, contact with the mother’s microbiota, hospital staff, and the hospital environment influences the type of microbial communities (mainly bacteria) that will colonize the various surfaces and tissues of the newborn [2,3,4,5]. Microorganisms are distributed in different communities across human skin, depending on their location. This promotes the establishment of an individual’s microbiome, which will consist of bacteria, parasites, fungi, and even phages and viruses [2,3,4]. Specifically, regarding the bacteriome of human skin, proteobacteria and actinobacteria tend to be the most predominant [4,5,6]. Species such as *Staphylococcus epidermidis*, several *Streptococcus* species, and *Lactobacillus* are abundant in childhood and subsequently decline, allowing the microbiome to be enriched with other bacterial genera. Therefore, their composition can vary at each life stage and be influenced by external conditions [4,5,6]. As commensals, the microorganisms that comprise the native microbiome of human skin play a crucial role in regulating the immune system in response to agents and stimuli that are potentially harmful to the human body [7].

A wound can be considered a loss of skin continuity [8,9]. Depending on the severity of the trauma, the skin may be affected at its outermost layer (epidermis), innermost layer (dermis), and even involve adipose or muscle tissue. This event triggers a series of physiological responses in the individual aimed at containing local tissue damage and subsequently regenerating it [8,9]. In the normal process of tissue repair, different cell lines are involved, participating in the activation of molecular mechanisms for the proliferation and migration of cells from the damaged skin epithelium (such as fibroblasts, endothelial cells, and keratinocytes). Likewise, the synthesis of extracellular matrix components for wound remodeling and the de novo creation of capillaries and blood vessels are part of this process [10,11]. The involvement of innate immune system cells throughout the physiological response is also essential, influencing the speed of re-epithelialization resolution by modulating the inherent inflammatory processes. Additionally, the microbial diversity that comprises the resident microbiome can affect the rate at which homeostasis is restored to the compromised skin layer [7,12]

**Figure 1:**
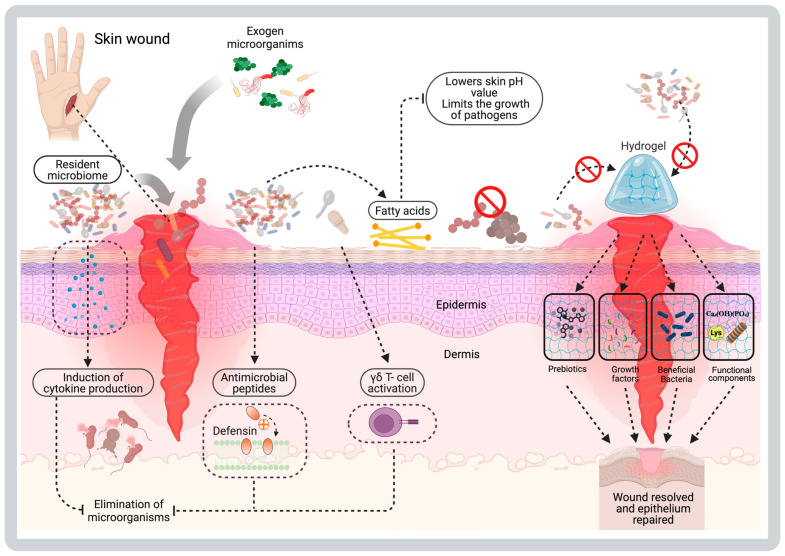
The importance of the microbiome and its interaction with the immune system in the tissue regeneration process, and the use of functionalized hydrogels to prevent infection of acute or chronic wounds.

In the case of chronic wounds, the healing process is affected, resulting in delayed tissue repair and an increased risk of microbiological infections. In this scenario, the affected skin tissue presents prolonged inflammatory processes, which can lead to local necrosis, impacting wound resolution [2,12,13]. As part of these physiological condition, the disruption of the resident microbiome generates dysbiosis and thus increases the risk of the formation of polymicrobial biofilms constituted by external pathogenic microorganisms (like *Pseudomonas aeruginosa*) and opportunistic resident microbiota (like *S*. *aureus*, *S. epidermidis* or *C. albicans*) that protect them against antibiotics and cellular immune response [7,12,14,15]. The detection of foreign microorganisms in wounds triggers persistent inflammation through an innate immune response mediated by cells of the skin tissue, which enhances microbiome imbalance and delays the healing process of the compromised skin barrier [7,12,14,15] (Figure 1).

Within microbial ecology, native skin microorganisms can utilize intrinsic mechanisms that enable them to occupy the available surface area and maintain their niches in balance. Strategies such as *quorum sensing* (*QS*), which is essential for communication in microbial communities, induce the formation of polymicrobial biofilms, the expression of antimicrobial proteins (such as bacteriocins), or the competition for resources needed to limit the growth of microorganisms foreign to the already established complexes [16,17]. Also, the microbiome can collaborate in response to the detection of other potentially pathogenic microbes that can cause wound infections. Activation of aryl hydrocarbon receptors in epidermal keratinocytes in response to the production of microbial metabolites promotes the differentiation of epithelial cells, thereby supporting the process of restoring skin integrity [6,18]. On the other hand, certain microorganisms that commonly are part of the human skin microbiome contribute to the hydrolysis of fatty acids, which modifies the skin pH to prevent the growth of potentially pathogenic exogenous microorganisms (such as *Staphylococcus aureus*) [2,3]. *Staphylococcus epidermidis*, like resident microorganisms, can produce enzymes with hydrolase activity that limit the invasion of potential pathogens.

Additionally, *S. epidermidis* can enhance the production of essential lipids, including those for sphingolipids, which are present in the skin barrier [16]. Another defense mechanism against potential pathogens stimulated by the resident microbiome is the production of antimicrobial peptides (AMPs) by keratinocytes. An example of this is human β-defensin 2 (hBD-2), a molecule expressed in response to the detection of fatty acids or the presence of bacterial structures. Some microbial residents are also capable of expressing AMPs to control bacterial growth or stimulate cellular responses with them [3]. Thus, it induces the expression of pro-inflammatory cytokines in the epithelial cells of the skin for the recruitment of innate immunity cells (such as neutrophils, dendritic cells, macrophages, and natural killer cells) involved in the management of infection processes in wounds [19,20,21,22,23].

Since the skin acts as a protective barrier, when its integrity is compromised, it opens the way to exogenous polymicrobial colonization, generating a disturbance in the native microbiome [4,24]. Furthermore, cutaneous microorganisms from the microbiome can access the wound site and trigger physiological processes related to their detection and containment. As the first line of defense, the innate immune system plays a crucial role in detecting the presence of microorganisms (resident or invasive) through pathogen-associated molecular patterns (PAMPs) and danger-associated molecular patterns (DAMPs) [25,26,27]. For the regulation of the inflammatory response, immune cells recognize various PAMPs through receptors (germline-encoded pattern-recognition or PRRs) such as Toll-like membrane glycoproteins (TLRs) or C-type lectins (CLRs), which detect microbial carbohydrates through recognition domains (CLDs) [25,26,27]. Additionally, there is evidence that microorganisms in the cutaneous microbiome, such as *S. epidermidis* or *Candida albicans*, participate in the immune response against potential infectious agents through the activation of T cells (γδ and helper 1/17, respectively) or the expression of various AMPs [3].

The microbiota plays a significant role in tissue regeneration, particularly in wound healing, which is often the initial step in more complex regenerative events: the gut microbiota, for example, has been shown to influence intestinal regeneration, studies on chronic wounds, such as diabetic foot ulcers, have provided valuable insights into the role of microbiota in wound healing [28]. Additionally, the bacterial species composition is a crucial factor in determining the timing and effectiveness of the regenerative process. The challenge for future research is to identify the specific roles of the microbiota and the signaling pathways by which they might modulate regeneration.

Ita has been seen that commensal bacteria influence immune responses, keratinocyte proliferation, and angiogenesis. *Staphylococcus epidermidis* promotes keratinocyte progression by upregulating Toll-like receptors (TLRs) and modulating TNF-α, which, through cutaneous CD8+ T lymphocytes, accelerates keratinocyte proliferation [29]. Studies conducted in male mice have demonstrated that prolonged intake of probiotics such as *Lactobacillus reuteri* not only reduces intestinal inflammation but also increases bone density and mass in vertebral bones [30].

Finally, it is important to highlight that in skin injuries, microbiota dysbiosis significantly influences the wound healing process. In this context, it has been demonstrated that the induction of dysbiosis markedly affects wound healing when compared to lesions in animals with a normal microbiota. Rodents with dysbiotic microbiota exhibit wounds of larger area and delayed healing compared to those with a balanced microbial composition [31].

In the search for strategies to promote tissue regeneration, the use of biomaterials that provide structural support, have a protective effect against infections, and modulate the resident microbiome has been evaluated. These are intended to encourage wound resolution and, consequently, the regeneration of the affected skin. Among biomaterials, hydrogels are synthesized from materials that are capable of polymerizing and absorbing water [32,33,34]. These polymers form a three-dimensional network that can emulate the physical properties of cellular tissues, making their use in wound healing a viable field [32,33,34]. Taking advantage of the absorption capacity of hydrogels, they can be used as a matrix for the controlled release of drugs, antibiotics and compounds with antimicrobial activity, as well as functionalized with proteins, cell growth factors or components of the extracellular matrix that are essential in the healing process of the damaged tissues [32,35,36]. In addition, the functionalization of hydrogels with prebiotics or probiotics can promote the reestablishment of the resident microbiome, as well as participate in the modulation of physiological responses related to immune and inflammatory processes (e.g., autoimmune diseases or implants) [34]. Hydrogels have been used to modulate the microbiome, for example, by applying a cationic hydrogel to modulate bacteria-related pro-inflammatory responses in the oral microenvironment. This approach decreased levels of LPS and cfDNA, downregulated TLR4/9-NFκB pathway activation, alleviated inflammatory alveolar bone loss, and potentially preserved the oral microbiome [37], suggesting that combining hydrogels and microbiome modulation is an emerging strategy for tissue regeneration.

This review aims to synthesize current knowledge regarding the application of functionalized hydrogels in restoring the balance of the tissue microbiome and their impact on wound healing and tissue regeneration. It will explore how these hydrogels leverage the body’s inherent microbial defense mechanisms and the host’s immune response to promote wound protection and physiological healing. The review will delve into the design and synthesis of functionalized hydrogels, highlighting their potential to modulate the microbiome through mechanisms such as the controlled release of antimicrobials, prebiotics, or probiotics, and their capacity to modulate inflammatory responses. Furthermore, the review will discuss clinical applications, challenges, and future perspectives of employing functionalized hydrogels in tissue regeneration, with a focus on restoring microbiome balance.

## 2. Design of Hydrogels with Antimicrobial and Modulatory Capacity

### Encapsulated Probiotics

Probiotic lactic acid bacteria play a vital role in maintaining gut health and microbial balance; however, their survival is significantly impacted by adverse environmental conditions, particularly low pH levels, which rapidly reduce their viability. To address this challenge, encapsulation strategies and protective delivery systems have been developed to enhance the stability of probiotics and maintain their beneficial effects. Chitosan and sodium alginate are among the most commonly used ingredients in encapsulation and hydrogel bead formulations due to their thickening and gelling properties. In this regard, *Lactobacillus plantarum* and *Lactobacillus rhamnosus* have been evaluated after encapsulation in alginate microbeads (180–260 µm in diameter) and exposure to a highly acidic environment (pH 2.0). These studies demonstrated high cell entrapment (95%) and viability (90%), with a uniform distribution of cells throughout the hydrogel matrix for *L. plantarum*, nonetheless, *L. rhamnosus* appeared to be less robust, exhibiting a rapid decline in viability [38], selectively reinforcing the effectiveness of encapsulation in preserving probiotic function under harsh conditions.

Another adverse condition is antibiotic exposure, which can promote bacterial resistance, reducing treatment effectiveness and complicating infection control. Using a two-step alginate cross-linking process, probiotics are encapsulated within alginate, a key component of *Pseudomonas* biofilms [39]. Encapsulation prevents tobramycin penetration into the hydrogel core, sequestering the antibiotic at the periphery while allowing probiotic metabolic byproducts to diffuse outward, exhibiting a four-log survival advantage over free probiotic and achieves complete eradication of methicillin-resistant *Staphylococcus aureus* (MRSA) and *Pseudomonas aeruginosa* in co-culture [39].

Another hydrogel matrix, composed of hyaluronic acid (HA) and cross-linked with extracellular polysaccharides (EPS-M76) from *Bacillus velezensis* M76T11B and dopamine, has been evaluated for its encapsulation efficiency and storage stability. The effects of cold storage (4 °C) on probiotic encapsulation demonstrate the hydrogel’s ability to preserve viability and stability over time and enhanced the lactic and acetic acid production, providing an effective encapsulation strategy for *Lactobacillus paracasei* TYM202 (HAEPS@*L.sei* gel) [40]. Despite storage conditions, the encapsulated probiotics remain metabolically active, ensuring their functional integrity. Additionally, EPS-M76 exhibits probiotic properties, acting as a prebiotic that supports bacterial proliferation within the hydrogel system, reinforcing its potential for biomedical applications [40].

Studies have evaluated calcium alginate-based beads incorporating Reishi mushroom extract for their prebiotic properties, protecting the viability of encapsulated *Lactobacillus acidophilus*. A single-layer vs. double-layer coating hydrogel formulation was compared to assess its stability and protective capacity. To further enhance stability and protect active ingredients during storage and gastro-intestinal conditions, the incorporation of maltose was found to significantly decrease the release rate of key bioactive molecules, including phenolic compounds, antioxidants, and β-glucan. Results confirmed that double-layer coated alginate hydrogel beads provided superior protection, ensuring better probiotic viability [41].

Inulin, a key prebiotic that promotes the growth of beneficial bacteria, when combined with alginate and collagen and encapsulated with a double chitosan coating (ACI-C), significantly boosted long-term probiotic viability; storage tests conducted at 4 °C for 105 days and 30 °C for 63 days showed that this ACI-C formulation outperformed sericin-coated alternatives. Moreover, the ACI-C system not only maintained superior survival rates under these conditions but also demonstrated enhanced resistance to simulated gastro-intestinal environments, improved thermal stability, and increased hydrophobicity [42]. Similarly, the superior prebiotic effect of inulin and apple marc flour in the alginate gel matrix during co-encapsulation not only enhanced bacterial viability but also resulted in larger particle sizes and increased tolerance to acidic environments. Furthermore, the type of lactic acid bacteria strains significantly influenced microcapsule size, with microcapsules containing *E. faecium* UAM18 being considerably larger than those formed with *A. viridans* UAM21B. Notably, these larger microcapsules, measuring approximately 100 µm, were associated with improved viability [43].

Fructooligosaccharides (FOS) extracted from banana peels have been used as a prebiotic encapsulation material for the delivery of *Lactobacillus rhamnosus* in combination with sodium alginate (SA). The microencapsulation process was evaluated under simulated gastro-intestinal conditions—such as exposure to bile salts, low pH, and extended storage—and significantly maintained probiotic viability. Among the formulations tested, the M2 formulation (50% SA + 50% FOS) demonstrated the highest encapsulation efficiency, whereas the M4 formulation (100% FOS) performed poorly, indicating that the prebiotic by itself is inadequate for effective encapsulation [44].

*Lactobacillus rhamnosus* GG (LGG) cells also has been microencapsulated in alginate beads with provitamin A hydrolysate achieving the goal of survival during storage (4 °C) with viability above 7 Log CFU/g by the end of 60 days of storage and in simulated gastric and intestinal juices, 77.4 and 8.5% after 120 min, respectively. In vivo, the administration of encapsulated LGG resulted in a reduction in both anaerobic bacteria and *Enterobacteriaceae* in rat feces, ensuring that the probiotic reached therapeutic levels in the colon. Interestingly, using the extrusion method for encapsulation, which produces microcapsules with a more uniform surface, proved to be superior to the emulsion technique (40). As previously reported, the emulsion microencapsulation technique may negatively affect viability, depending on the type of homogenizer employed [45].

Although it has been reported that prebiotic components can enhance cell viability, Chavarry et al. demonstrated that encapsulating the prebiotic quercetin with *Lactobacillus gasseri* or *Bifidobacterium bifidum* in chitosan-coated alginate microspheres resulted in only a minimal yield of viable cells. Furthermore, the survival of these probiotics during storage at 4 °C was poor, indicating that the inclusion of quercetin compromised bacterial viability. In contrast, when quercetin was not incorporated, the encapsulated bacteria exhibited resistance to simulated gastric conditions (pH 2.0 for 2 h) and to a 3% bile solution (for 2 h), significantly improving their survival [46].

In experiments using transglutaminase (TGase)-cross-linked gelatin (GE) and sodium hexametaphosphate (SHMP) hydrogels, a dense, low-porosity network is formed, resulting in minimized release of encapsulated *L. plantarum* under simulated gastro-intestinal conditions. Specifically, in simulated gastric juice (0.85% saline at pH 2.0 with pepsin) and simulated intestinal fluid (0.85% sterile saline at pH 6.8 with bile salts and pancreatic enzymes), the encapsulated bacteria achieve survival rates between 75% and 95%. Notably, bacterial viability initially improved with increasing enzyme concentration, but then declined at higher levels. Moreover, the use of advanced drying techniques—such as microwave vacuum freeze-drying (MFD)—further enhanced the stability of the microcapsules and improved bacterial survival over 28 days of storage at 4 °C [47].

Likewise, the viability of *Lactobacillus plantarum* during both digestion and storage can be significantly enhanced by incorporating gelatin into alginate to produce alginate-gelatin (ALG-GE) hydrogel beads. Results confirmed the successful incorporation of probiotics into the beads, exhibiting an encapsulation efficiency of 97.7% and concurrent improvements in storage stability (15%) and thermal stability (8%). These beads acted as a protective barrier, preventing the probiotics from being deactivated in simulated gastric fluid while later facilitating their controlled release in simulated intestinal fluid [48].

## 3. Customizing Hydrogel Methods

### 3D Bioprinting Techniques to Customize the Shape and Hydrogel Gelation via UV Irradiation or Thermal Self-Assembly

**Figure 2:**
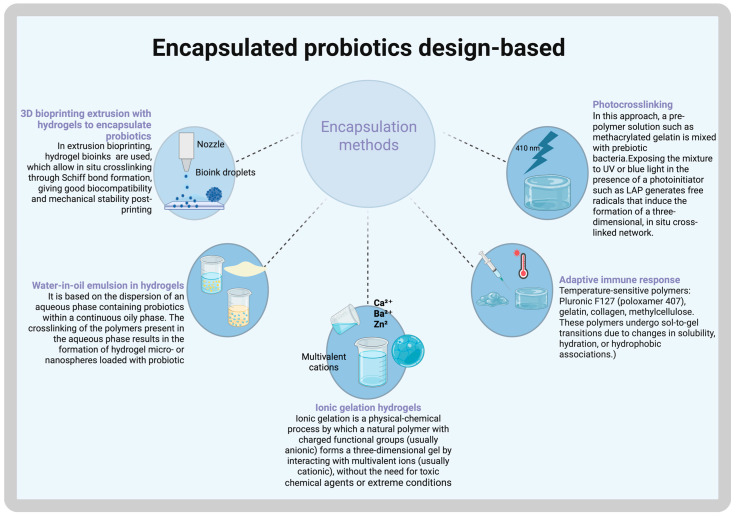
Schematic representation of encapsulation techniques used to protect probiotic cells, employing materials such as alginate, chitosan, and proteins. These systems enhance cell viability, stability under adverse conditions, and targeted release within the gastrointestinal tract or wound sites. In wound healing, encapsulated probiotics contribute to tissue regeneration, infection control, and modulation of inflammation through sustained delivery and improved microbial viability.

Three-dimensional (3D) printing is an advanced technology that enables the fabrication of customizable medical products [49]. Unlike traditional production methods, where pore size, pore distribution, and scaffold architecture are difficult to control, 3D printing allows for precise customization of these structural parameters. However, not all biomaterials are suitable for 3D printing, and successful printing in the vertical dimension depends on the bonding strength between layers to ensure structural integrity [49]. For tissue regeneration, the conservation of all structural properties of hydrogels is key. Other methods for encapsulating probiotics within hydrogels are shown in Figure 2.

The viscoelastic properties of digested decellularized extracellular matrix (dECM) in acidic solutions play a critical role in its suitability as a bio-ink for 3D printing. For instance, the application of 0.1 M hydrochloric acid enhances the digestion rate, leading to the formation of a softer dECM hydrogel with reduced stiffness. However, this decrease in mechanical strength poses a challenge in maintaining the stability of multilayer (layer-by-layer) scaffolds, which is essential for structural integrity in 3D-printed biomaterials [50].

To enhance the mechanical properties and stability of bio-inks for 3D printing, cellulose nanofibrils (CNF) have been incorporated as a reinforcement in alginate-based composites to improve structural integrity. To further optimize the formulation, glycerin has been added to reduce volatile components that contribute to instability at room temperature, thereby preventing evaporation and enhancing the material’s stability. However, the hydrogel can lose its shape over time, requiring ionic cross-linking with CaCl2 to improve its structural stability [51].

In response to this challenge, self-healing hydrogels have emerged as an innovative solution capable of autonomously restoring structural integrity after deterioration without requiring external intervention. They ensure durability while preserving their original properties, relying on dynamic covalent bonds that can reversibly adapt and reorganize. One example is the printable self-healing hydrogel SPBC, which is based on a poly (vinyl alcohol) (PVA), sodium alginate (SA), and cellulose nanofiber (CNF) matrix. This material is cross-linked with sodium tetraborate through hydroxyl groups present on CNF, enabling rapid self-healing and support for 3D printing [52].

In this way, gelatin biomaterial has been enhanced with methacrylate as a cross-linker in Gelatine Methacryloyl (GelMA) hydrogels, enabling injectability and tunability. Additionally, this process facilitates in situ polymerization through photo-crosslinking under ultraviolet (UV)-visible light irradiation, making them suitable for applications in irregular wounds and the development of wound dressings [53]. Similarly, leveraging photothermal therapy (PTT), injectable photosensitive antibacterial complex hydrogels have been developed. This approach utilizes a light-responsive material activated by near-infrared (NIR) light to efficiently eliminate bacteria by generating heat through localized hyperthermia or the production of reactive oxygen species. However, excessive temperature may exceed healthy tissue tolerance, potentially causing cell damage [54].

Biomaterials and cross-linking methods for probiotic/prebiotic delivery are presented in Table 1.

## 4. Mechanisms of Microbiome Modulation

### 4.1. Biofilm Inhibition and Antimicrobial Effect Through Hydrogels

Biofilms are intricate bacterial communities, consisting of multiple species that adhere to surfaces, such as tissues. These clusters are embedded within a self-produced extracellular polymeric substance (EPS) matrix, forming protective, gel-like coatings that enhance bacterial survival [64].

**Figure 3:**
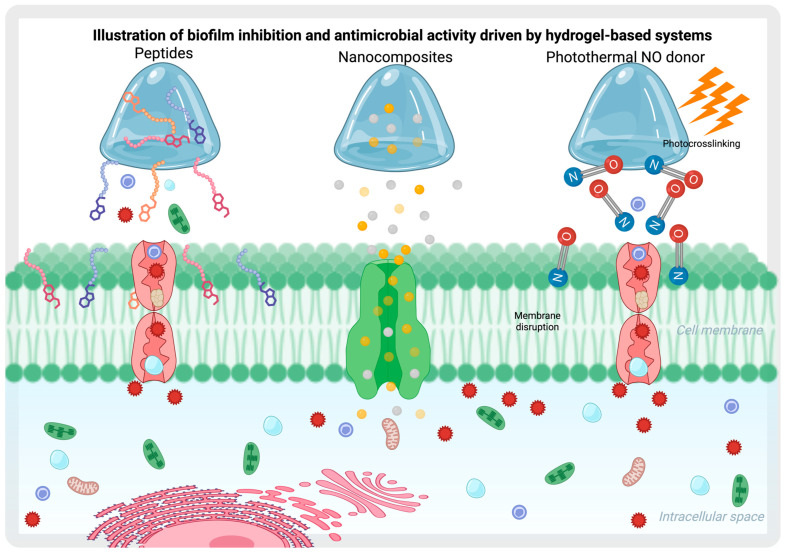
Antimicrobial peptides disrupt bacterial membranes and interfere with biofilm matrix integrity. Nanoparticles penetrate the extracellular polymeric substance (EPS), delivering agents that induce oxidative stress and gene regulation. Thermosensitive compounds release nitric oxide upon temperature-triggered activation, promoting bacterial dispersal, immune modulation, and enhanced wound healing.

A multi-functional injectable hydrogel has been developed for the eradication of bacterial biofilms and wound healing, utilizing a near-infrared (NIR) light-responsive nano-platform (PSPG hydrogel). This system integrates platinum (Pt)-decorated gold nanoparticles and employs a sodium nitroprusside (SNP)-loaded porphyrin metal-organic framework (PCN) as an inner template. The PSPG hydrogel continuously scavenges endogenous H_2_O_2_ via its incorporated Pt nanozyme, thereby enhancing local oxygen concentrations to promote effective sterilization and biofilm disruption. Through the synergistic effects of photodynamic therapy (PDT) and photothermal therapy (PTT), this hydrogel ensures comprehensive antimicrobial action and accelerated wound healing [65] (Figure 3).

Alternative strategies utilize nitric oxide (NO) to disrupt bacterial membranes, DNA, and proteins. As an antimicrobial agent, NO penetrates biofilms effectively and generates reactive nitrogen species (RNS) to inactivate bacterial cells. A quaternized chitosan (QCS)-based hydrogel, designed as a multi-functional system with photothermal near-infrared (NIR)-mediated NO donors, incorporated N,N′-di-sec-butyl N,N′-dinitroso-1,4-phenylenediamine (BNN6) in mesoporous polydopamine (MPDA). The photothermal effect contributes to the dispersal of biofilms, leading to the efficient eradication of *S. aureus* biofilms in both severely infected in vitro and in vivo wound models [66]. Additionally, L-arginine-modified photonic chitosan hydrogels, loaded with a novel indocyanine green formulation (PG@Arg/IR820), facilitate the generation of reactive oxygen species (ROS). The resulting ROS further oxidizes L-arginine, a natural source of NO, triggering the release of NO for gas therapy [67]. Moreover, an injectable gelatin-based hydrogel has been developed as an NO-releasing system by incorporating S-nitrosothiolated gelatin. This formulation exhibits potent antibacterial activity, as demonstrated in tests with *Escherichia coli* and *Staphylococcus aureus*, highlighting its high bactericidal potential [68].

Among other emerging strategies, silver nanoparticles (AgNPs) have demonstrated potent biofilm-disrupting capabilities by targeting the exopolysaccharide matrix, leading to structural destabilization and interference with peptidoglycan components in bacterial cell walls. At an intracellular level, AgNPs induce physical damage, ion release, and oxidative stress, promoting bacterial inactivation through the production of reactive oxygen species (ROS) and DNA disruption [69]. The development of silver nanoparticle-loaded hydrogels represents a multi-functional strategy for eradicating biofilms, reducing microbial resistance, and promoting tissue regeneration. Some gelatin-based hydrogels loaded with chitosan-stabilized AgNPs exhibit anti-biofilm activity against *Staphylococcus aureus*, *Bacillus subtilis*, *Pseudomonas aeruginosa*, and *Escherichia coli* [70]. Additionally, studies have shown that chitosan-stabilized AgNPs and AuNPs demonstrated biofilm inhibition rates of 53.21% and 79.39% for *P. aeruginosa* and 48.71% and 48.16% for *S. aureus*, respectively. However, chitosan stabilization improved the biofilm-disrupting activity of AuNPs but did not enhance the efficacy of AgNPs. Interestingly, for *S. aureus*, no significant improvement was observed in either nanoparticle system [71].

Additionally, Gelatin-based hydrogels incorporating bio-nanosilver AgNPs functionalized with lactoferrin (LTF) were explored as dual-antimicrobial systems, leveraging LTF’s iron-chelating properties to enhance further anti-biofilm activity against *S. aureus* and *P. aeruginosa* [72]. Similarly, a chitosan-stabilized silver nanoparticle (AgNP), lactoferrin (LTF), and Dicer-substrate short interfering RNA (DsiRNA)-complexed hydrogel formulation was investigated for its anti-biofilm effects against both Gram-positive and Gram-negative bacteria. In vitro experiments demonstrated significant biofilm inhibition of *P. aeruginosa* and *S. aureus*, as indicated by crystal violet staining, along with a reduction in the number of viable bacterial cells within established biofilms following exposure to the treatment. Notably, LTF’s iron-chelating activity further enhanced biofilm suppression compared to hydrogels containing AgNPs alone [73]. An alternative approach leveraging deferoxamine (DFO), a potent iron chelator, offers a similarly effective strategy for biofilm eradication. DFO selectively sequesters free iron, disrupting bacterial metabolism and weakening biofilm integrity, thereby impairing the formation of MRSA biofilms. To further enhance biofilm clearance, MXene nanoparticles are incorporated into DFO-loaded hydrogels, introducing a photothermal effect under near-infrared (NIR) irradiation. MXene efficiently converts light into heat, destabilizing biofilms and increasing antimicrobial efficacy [74]. Additionally, tannic-acid-stabilized silver nanoparticles (TA-AgNPs) embedded within an alginate hydrogel also demonstrated potent antimicrobial efficacy against polymicrobial wound biofilms derived from *Streptococcus pyogenes*, *Staphylococcus aureus*, and *Pseudomonas aeruginosa*, achieving a 3–4 log reduction in bacterial populations [75].

Beyond the functionalization of hydrogels with antimicrobial agents, it has been demonstrated that some hydrogels inherently exhibit anti-biofilm activity, even without the incorporation of additional antimicrobial agents These materials disrupt biofilm formation through mechanisms such as surface interactions, hydration effects, and local environmental modifications, making them valuable for biomedical applications (Table 2) [76].

Among these, low-viscosity chitosan has proven to be an effective standalone biofilm inhibitor, significantly reducing the formation of *Staphylococcus epidermidis* biofilms. When introduced into growth media or coated onto polystyrene surfaces, its anti-biofilm effect is concentration-dependent, with greater efficacy observed at 0.5% and 1% *w*/*v* [76]. Also, native medium molecular weight chitosan medium treatment on mature biofilms can strongly inhibit viable *Listeria monocytogenes* cells, achieving a reduction of approximately 6 logs. It also produced 3–5 log decreases in the biofilms of *Bacillus cereus*, *Salmonella enterica*, and *Pseudomonas fluorescens*, while its effect on *Staphylococcus aureus* was less pronounced [77]. Similarly, low-molecular-weight chitosan demonstrated an anti-biofilm effect on polyurethane-like catheters by significantly reducing the metabolic activity of both *S. epidermidis and C. albicans* (64), as well as in an in vivo model [79].

Carboxymethyl-chitosan also demonstrated a strong inhibitory effect not only on monomicrobial biofilms but also on polymicrobial biofilms of *S. epidermidis* and *C. tropicalis*. This inhibition was confirmed through assays performed on both microplates and silicone surfaces, with its effectiveness sustained over short and extended periods. [80] A chitosan hydrogel containing the natural antimicrobial peptide epsilon-poly-L-lysine was tested ex vivo on mature polymicrobial wound biofilms composed of *Pseudomonas aeruginosa*, *Staphylococcus aureus*, and *Candida albicans*. The topical gel reduced biofilm thickness by 84% within 24 h [81]. Furthermore, the formulation demonstrated in vivo effectiveness by significantly reducing *Pseudomonas aeruginosa* biofilms in a murine burn wound infection model [84].

**Figure 4:**
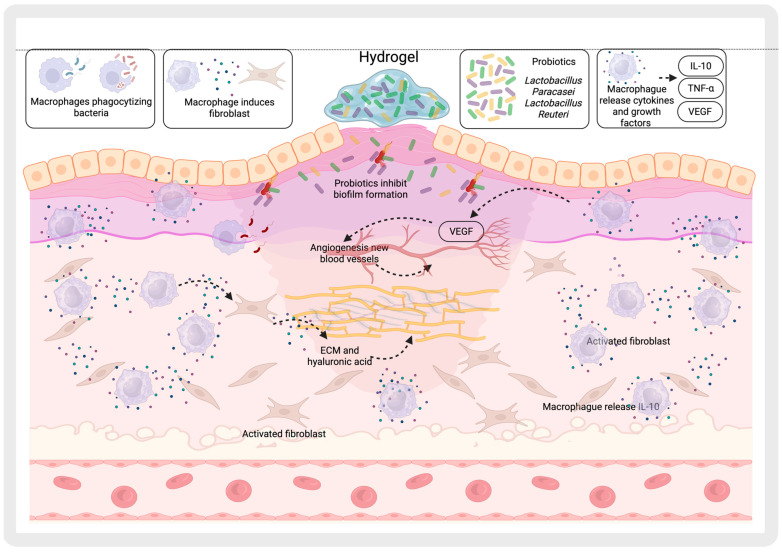
Anti-inflammatory mechanisms triggered following tissue injury and subsequent administration of probiotic-loaded hydrogels involve the release of bioactive metabolites that promote angiogenesis through the upregulation of growth factors such as vascular endothelial growth factor (VEGF). In parallel, macrophages are activated and contribute to tissue repair by stimulating fibroblasts, which in turn secrete new extracellular matrix components, including collagen and hyaluronic acid, thereby supporting tissue remodeling and regeneration.

Chitosan-coated surfaces have been shown to possess potent anti-biofilm properties by reducing the viability of biofilm cells from *Klebsiella pneumoniae*, *Pseudomonas aeruginosa*, and *Candida albicans* by up to 99.9997% [82]. And chitosan chemically modified into Chitooligosaccharides (COS) slightly inhibits *S. aureus* biofilm formation [83]. This intrinsic antimicrobial property highlights chitosan’s potential as a self-sufficient biofilm-disrupting agent, offering promising applications in medical coatings, wound dressings, and infection control strategies. In this regard, materials designed to inhibit biofilm formation while promoting beneficial immune responses can be supported by various mechanisms. For example, the encapsulation of bacteria within hydrogels does not negatively impact the comensal microbiota. Moreover, when these hydrogels exhibit pH sensitivity and release antibiotics selectively in alkaline environments—such as those present in infected wounds—they do not harm commensal bacteria [85]. Additionally, it has been demonstrated that the localized release of probiotics such as *Lactobacillus reuteri* in inflamed tissues, mediated by the selective degradation of ROS-responsive hydrogels, preserves the biofilm in non-inflamed regions (Figure 4) [62].

Another mechanism involves the use of hydrogels containing microbial extracellular polysaccharides that encapsulate *L. paracasei*, promoting inflammatory regulation, angiogenesis, and microbial homeostasis, while also facilitating the establishment of beneficial microbiota [40]. Finally, the combination of these probiotics with anti-inflammatory agents has been shown to modulate the TLR4/MyD88/NF-κB signaling pathway, effectively reducing inflammation without disrupting the beneficial biofilm [86].

Also, it been demonstrated EcN administered intragastrically at doses of 10^8^ CFU/kg for EcN and 70 mg/kg for GPM, every other day for a total of three doses restored body weight, colon length and shifted the gut microbiota composition toward that of healthy controls, highlighting its microbiota-modulating potential in vivo treatment [81].

### 4.2. Promotion of Beneficial Microbiota

An experimental model replicating bacterial growth within a biofilm-like microenvironment has demonstrated intra-species communication among spatially confined *E. coli* OR colonies encapsulated within a natural alginate hydrogel matrix. This system supports the long-term viability of complex microbial subpopulations, sustaining their interactions for over 10 days under physiologically relevant conditions, analogous to those found in biofilms. QS is activated as cell density increases, leading to the induction of nisin production when *Lactococcus lactis* strains are co-cultured. Moreover, stratified communication patterns were observed, highlighting the role of hydrogel beads as a highly effective medium for facilitating the transport of signaling molecules within the encapsulated bacterial communities [87].

**Figure 5:**
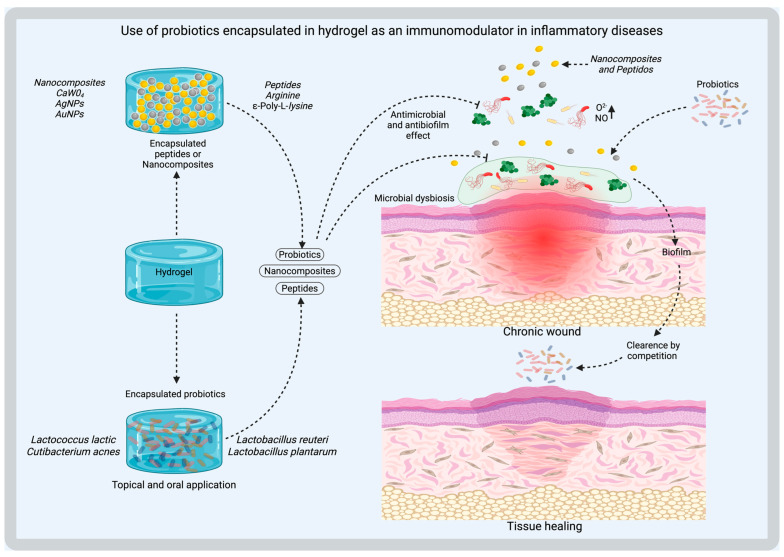
The probiotic hydrogel’s regulatory role, along with the composite’s antimicrobial and antibiofilm properties, plays a key role in restoring balance to microbiota dysbiosis. The composite exerts its effects via distinct mechanisms, including disruption of the exopolysaccharide matrix, enhancement of oxygen and nitric oxide availability, and facilitation of microbial clearance through competitive exclusion.

Figure 5 shows a multi-functional calcium tungstate (CaWO_4_) nanoparticle-loaded alginate microgel (CTM)-based system for oral probiotic delivery. Released tungsten can inhibit the growth of Enterobacteriaceae by attenuating Enterobacteriaceae-dependent molybdenum enzyme activity. Since the development of probiotics is independent of molybdenum enzymes, tungsten has no significant effects on probiotics loaded in CTM. Consequently, CTM can selectively suppress the growth of Enterobacteriaceae in colitis, thereby disrupting the ecological niche occupied by pathogenic bacteria and promoting the colonization of probiotics. Using *Bacillus coagulans* (BC) as a candidate probiotic, the authors demonstrated that orally delivered BC-containing CTM (i.e., BC@CTM) effectively inhibited the proliferation of harmful bacteria, facilitated probiotic colonization, and restored gut microbiota homeostasis in mice with dextran sulfate sodium (DSS)-induced colitis [56,88]. Other biomaterials based on yeast cell wall (YCW) to encapsulate CaWO_4_ also regulate the gut microbiome by specifically suppressing the abnormal expansion of *E. coli* during colitis and boosting probiotic growth [89].

Probiotic *Escherichia coli* Nissle 1917 (EcN), attached to gelated macrophages encapsulated within poly(ethylene glycol) diacrylate (PGE-DA) (GPM-EcN), can prolong intestinal retention time after oral administration, leading to an improvement in bacterial diversity and a significant shift in the microbiota composition in rats with inflammatory bowel disease (IBD). Treatment with GPM-EcN effectively restored the elevated Firmicutes/Bacteroidota ratio to normal levels, contributing to microbiota balance and potential therapeutic benefits [90].

A hydrogel patch was created by embedding photothermal bacteria, a photosensitizer, and a reactive oxygen species amplifier in a protein hydrogel. The bacteria can express melanin granules, giving them a photothermal response when exposed to NIR-II laser irradiation. Tetramethylpyridinium porphyrin on the bacterial surface and laccase in a protein gel can produce singlet oxygen and hydroxyl radical when exposed to visible light and lignin, respectively. The engineered bacteria hydrogel patch was effective in photothermal, photodynamic, and chemodynamic therapy, treating bacterial infections in mouse wounds and improving wound healing [57].

## 5. Interaction with the Immune System

### Regulation of T Cells and Reduction in Immune Responses Through Hydrogels with Modified Bacteria

Current research focuses on hydrogel-based encapsulation of genetically modified bacteria as a strategy to minimize immune responses. *Escherichia coli* encapsulated in Pluronic F127-based hydrogels with enhanced cross-linking effectively prevents bacterial escape and physical interaction between bacteria and immune cells in culture. This encapsulation approach reduces activation and alters the differentiation of NK cells, CD4+ T cells, and CD8+ T cells, while still inducing some release of pro-inflammatory cytokines (IL-6 and IFN-γ) from normal peripheral blood mononuclear cells (PBMCs). However, the observed cytokine levels remain comparable to those induced by empty hydrogels in pro-inflammatory PBMCs, suggesting minimal additional immune stimulation [91].

**Figure 6:**
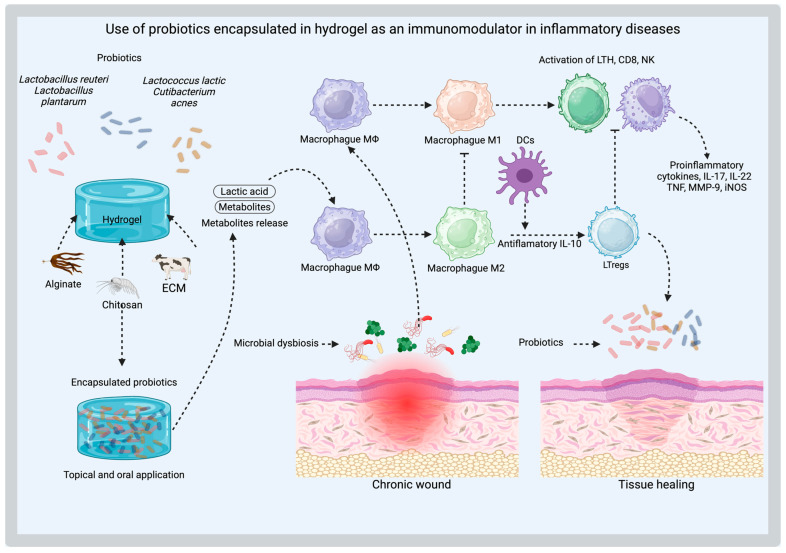
The probiotic-mediated defense and the underlying biological mechanism that supports the wound healing potential of the hydrogel. Lactic acid and related metabolites induce anti-inflammatory macrophage polarization via IL-10 secretion and LTregs activation, thereby reversing the pro-inflammatory state and suppressing lymphocyte activity.

In the case of probiotics, they can regulate intestinal immune responses by stimulating immune cell activity, promoting the growth of beneficial bacteria, and protecting against pathogenic invasion. They also help to maintain gut microbiota balance and preserve intestinal barrier integrity, contributing to overall digestive health [92]. Nonetheless, specific probiotic strains, such as *Lactobacillus reuteri* and *Lactobacillus casei*, but not *Lactobacillus plantarum*, exhibit immunomodulatory properties by priming monocyte-derived dendritic cells (DCs) to facilitate the development of T regulatory (Treg) cells. These regulatory T cells (Tregs) produce elevated levels of IL-10, enabling them to suppress the proliferation of bystander T cells in an IL-10–dependent manner, contributing to immune balance and anti-inflammatory responses (Figure 6) [93].

Meanwhile, nanoencapsulation of probiotics and the anti-inflammatory agent 5-aminosalicylic acid (5-ASA) within gastro-intestinal microenvironment-responsive calcium alginate polysaccharides have been shown to offer synergistic therapeutic effects. This approach enhances microbiota richness and diversity, reduces the expression of pro-inflammatory cytokines, and helps to restore intestinal barrier integrity, contributing to effective colitis management [94].

A heparin-poloxamer (HP) hydrogel incorporating engineered *Lactococcus lactis* has been proposed for VEGF secretion, leveraging its natural ability to produce lactic acid as a signaling metabolite. This feature facilitates M1 macrophage polarization into M2-like phenotypes, enhancing the expression of CD206 and ARG1, while simultaneously reducing the expression of inflammatory mediators, including TNF-α, nitric oxide, iNOS, and MMP9 proteases. This modulation induces an anti-inflammatory microenvironment, promoting wound healing in diabetic patients [58].

Recent advancements in injectable hydrogel formulations have led to the development of a self-healing hydrogel, combining oxidized konjac glucomannan (OKGM) and arginine-modified chitosan (CS-Arg) (OC) with protocatechualdehyde-@Fe (PF) (OC/PF). This bioactive material has shown significant antibacterial and anti-inflammatory effects in methicillin-resistant *Staphylococcus aureus*-infected full-thickness mouse wounds. The hydrogel not only accelerates tissue repair but also plays a key role in immune modulation. It encourages M2 macrophage polarization, which helps mitigate inflammation while promoting tissue regeneration [95].

Other strategies aim to provide an alternative to live bacteria therapy by employing bacterial components. For example, in the treatment of psoriasis, a chronic inflammatory skin disease influenced by dysregulated skin microbiota, it has been proposed to use extracellular vesicles derived from microorganisms. In this approach, *Cutibacterium acnes*-derived extracellular vesicles (CA-EVs) are encapsulated in gelatin methacrylate (GelMA). This formulation effectively inhibits the transition of cutaneous-resident innate lymphoid cells type 2 (ILC2) into pathological ILC3 cells, suppresses the secretion of pro-inflammatory cytokines IL-17 and IL-22, and helps restore epidermal barrier function while maintaining skin microbiota homeostasis [59]. Similarly, a subcutaneous injection of a thermosensitive PF-127 hydrogel loaded with *Parabacteroides goldsteinii*-derived outer membrane vesicles (Pg OMVs) carrying pentadecanoic acid has been proposed for ameliorating symptoms of psoriasis. This system not only provides controlled, sustained release of the bioactive vesicles but also enhances their ability to reduce inflammatory cell infiltration, inhibit keratinocyte hyperproliferation, and temper overactivated immune responses through modulation of the IL-23/Th17 axis [60]. Meanwhile, the HAEPS@L.sei gel demonstrated a significant increase in M2 macrophages on wound healing, with serum levels of IL-10 and VEGF-α markedly elevated and TNF-α notably reduced. This response was attributed to the lactic acid released by the probiotic, highlighting its role in enhancing the body’s anti-inflammatory response and tissue repair processes [40].

It has also been reported that *Bacteroides fragilis*-derived outer membrane vesicles (OMVs) secrete the polysaccharide A (PSA) capsular antigen. PSA is recognized via TLR2 on dendritic cells (DCs), which in turn activates Gadd45a signaling. This cascade leads to the production of the immunoregulatory cytokine IL-10 by both DCs and regulatory T cells (Tregs), ultimately preventing T helper cell proliferation and the production of inflammatory mediators [96]. Although incorporating these bacterial components into a hydrogel-based delivery system has not yet been reported, it presents a promising concept for a novel therapeutic strategy to treat inflammatory conditions.

A more focused strategy utilizes the specific application of the bacteriocin Jileicin, integrated into a hyaluronic acid/gelatin-based multi-functional bioadhesive hydrogel. Jileicin directly enhances the phagocytic and bactericidal capabilities of macrophages. In vivo, treatment of infected skin defects in diabetic mice resulted in M2-type macrophage polarization, which inhibited inflammation and promoted wound healing, further enhancing the bacterial membrane-disrupting effect against methicillin-resistant MRSA [97].

Furthermore, peritoneal macrophages were subjected to intracellular hydrogelation with PGE-DA to produce nonviable gelated peritoneal macrophages (GPMs). This process preserves membrane receptors and maintains an intact membrane structure, thereby enhancing cell interactions. The preserved receptors on the GPM membranes specifically recognized LPS on the probiotic *Escherichia coli* Nissle 1917 (EcN) via TLR4, forming the GPM-EcN conjugate without affecting bacterial proliferation or triggering macrophage activation. It resolved the clinical challenge of probiotics being rapidly eliminated with oral administration Additionally, the presence of tumor necrosis factor receptor 2 (TNFR2) and interleukin-1 receptor type 2 (IL1R2) on GPM facilitated the binding and neutralization of cytokines such as TNF-α and IL-1β, without triggering M1 polarization, and neutralized LPS-driven inflammatory responses in intestinal epithelial cells [90].

Below is a table with a summary of biomaterial-based systems for immune modulation: in vivo evidence (Table 3).

## 6. Applications in Tissue Regeneration and Infection Control

### 6.1. Chronic Wounds

The economic and social burdens of chronic wounds have a significant impact on the global health system, representing a substantial challenge [98]. One of the primary approaches in tissue regeneration focuses on the formation of new extracellular matrix, predominantly composed of collagen. Based on this principle, numerous hydrogels with varying compositions have been developed to address impaired wound healing [99].

In patients with diabetes, these conditions are exacerbated by increased local hyperglycemia, susceptibility to infection, and oxidative stress. Adenine- and thymine-modified chitosan hydrogels, coupled with S-nitrosoglutathione (GSNO) and binary L-arginine (bArg), through the release of nitric oxide, have demonstrated favorable results in chronic wound healing. This hydrogel promotes the expression of HIF-1*α* and VEGF. Histological and immunohistochemical staining show denser neurovascularization and increased collagen deposition. In addition, this hydrogel demonstrated bacteriostatic capacity against Escherichia coli and Staphylococcus aureus, indicating the potential of the hydrogel [100].

Among the various formulations available for hydrogels, those composed of chitosan and hydroxypropyl methylcellulose supplemented with insulin have also shown favorable outcomes. When evaluated in diabetic C57BL/6 mouse models (characterized by blood glucose levels exceeding 250 mg/dL), these hydrogels demonstrated accelerated wound closure, enhanced tissue organization, and hair follicle regeneration within 20 days. The regenerative properties of the hydrogel are attributed to the inclusion of insulin, as it directly regulates the growth of human dermal cells [101].

Alternative regenerative strategies target mitochondrial function as a key pathway in tissue repair. The regenerative potential of adult stem cells is known to depend mainly on the paracrine secretion of mitochondrial extracellular vesicles. In this context, microneedle-array hydrogel patches have shown promise in promoting tissue regeneration and wound healing via mitochondrial mechanisms. When functionalized with metformin to enhance mitochondrial biogenesis, these hydrogels stimulate macrophage polarization toward the M2 phenotype, associated with anti-inflammatory and regenerative functions, thereby accelerating the healing of radiation-induced wounds in murine models within 14 days of treatment [102].

### 6.2. Hydrogel Coatings with Hydroxyapatite to Prevent Infections in Bone Prostheses

Screws have been widely used for prosthesis fixation in orthopedic surgery with relative success; however, their effectiveness largely depends on the quality of the surrounding bone. To reduce the risk of failure, materials such as hydroxyapatite, calcium phosphate, and polymethyl methacrylate bone cements have been investigated as potential reinforcement strategies [103].

Hydroxyapatite is widely regarded as an ideal material for repairing bone defects due to its excellent biocompatibility, osteoinductivity, osteoconductivity, and bioactivity. The incorporation of metal ions such as strontium has been shown to enhance their biological and antimicrobial properties. Inhibition zone assays have demonstrated improved antibacterial activity against *E. Coli* and *S. Aureus* following strontium doping [104].

In the context of antibacterial hydrogels, recent studies highlight the potential of mimicking the innate antibacterial defense mechanism of the *Chinese alligator*, *Andrias davidianus*, as a viable strategy for controlling bone infections. Furthermore, the incorporation of hydroxyapatite enhances the mechanical stiffness of the hydrogel, making it suitable for filling bone defects. Both in vitro and in vivo evaluations demonstrate that this hydrogel effectively mitigates Staphylococcus aureus-induced bone infections, preventing the progression of osteomyelitis [105].

## 7. Hydrogels Modulating the Microbiome: Suggested Uses in Preclinical and Clinical Studies

### 7.1. Hydrogels of Prevotella Histicola for Rheumatoid Arthritis

**Figure 7:**
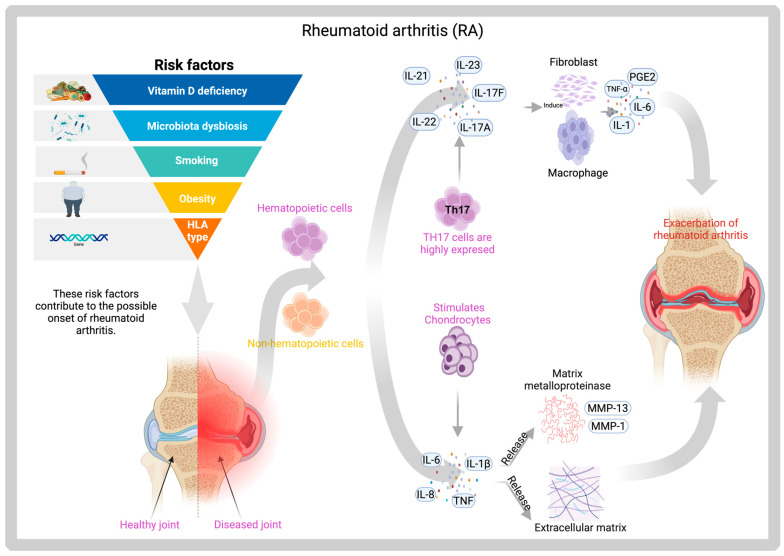
Rheumatoid arthritis (RA) is a chronic inflammatory disease influenced by multiple risk factors, including vitamin D deficiency, smoking, microbiota dysbiosis, obesity, and genetic predisposition (particularly certain HLA types). These factors contribute to the transition from healthy to a diseased joint, where both hematopoietic and non-hematopoietic cells become involved. Th17 cells are highly expressed in RA and produce pro-inflammatory cytokines, including IL-17A, IL-17F, IL-21, IL-22, and IL-23, which stimulate macrophages and fibroblasts to release additional mediators, such as TNF-α, IL-1, IL-6, and PGE2. Simultaneously, chondrocytes are activated to secrete IL-6, IL-8, IL-1β, and TNF, further amplifying inflammation. This inflammatory environment promotes the release of matrix metalloproteinases (MMP-1 and MMP-13), which degrade the extracellular matrix, leading to cartilage destruction and joint damage. Together, these mechanisms drive the progression and exacerbation of RA.

Rheumatoid arthritis (RA) is the most common autoimmune joint disorder worldwide, affecting approximately 0.5–1% of the global population. The primary clinical manifestations of RA include minor joint pain in the hands, wrists, and feet, functional impairment, swelling, and deformity. Additionally, the disease can affect multiple organs and systems, significantly compromising patients’ quality of life [106,107]. The disease primarily targets the extracellular matrix of articular cartilage. Matrix metalloproteinases (MMPs), a family of zinc-dependent endopeptidases, play a key role in the degradation of the extracellular matrix during disease progression (Figure 7). Among emerging therapeutic strategies for RA, hydrogels have gained attention as a viable platform. When combined with bioactive molecules (BMs), these hydrogels exhibit enhanced therapeutic efficacy [108].

In patients with RA, interactions with the gut microbiota have been closely linked to the onset and progression of the disease. The bacterial genus Prevotella plays a complex role in both the improvement and progression of the condition [109]. *Prevotella histicola* is an anaerobic bacterium that resides in the gastro-intestinal tract and has been shown to suppress autoimmune encephalomyelitis in murine models [110]. Clinical studies in patients with RA have reported gut microbiota dysbiosis, characterized by a marked reduction in species from the Bacteroides–Porphyromonas–Prevotella group when compared to healthy controls. In vivo studies using HLA-DQ8-susceptible mice models of RA have shown that oral administration of *Prevotella histicola* leads to a significant reduction in pro-inflammatory cytokines, including IL-2, IL-17, and TNF-α. After seven days of treatment, disease progression in the treated group was notably attenuated compared to control mice, further supporting the therapeutic potential of *P. histicola* in modulating immune responses associated with RA [111].

*P. histicola* has demonstrated reparative effects in AR when administered in combination with a tumor necrosis factor (TNF) inhibitor in DQ8 mouse models [109,112], additionally, other studies employing similar methodologies have reported a decreased incidence of RA as a result of reduced serum levels of cytokines such as IL-2, IL-17, and TNF-α. These effects were accompanied by an increase in regulatory T cells in the intestinal tissue of mice and a corresponding reduction in antigen-specific Th17 responses [113].

Further evidence supporting the critical role of *Prevotella histicola* is provided by proteomic analyses conducted using bioinformatics approaches. Results have shown that particular bacterial species, including Bacteroides plebeius, Bifidobacterium bifidum, Lactobacillus casei, and *Prevotella histicola*, were significantly reduced in patients with RA [114].

Hydrogels represent a promising platform to enhance the outcomes of microbiota-based interventions in rheumatoid arthritis (RA), particularly those involving the therapeutic use of *Prevotella histicola*. However, the effective delivery and colonization of this obligate anaerobe remain a major challenge. Hydrogels, due to their highly tunable physical and chemical properties, can address these limitations by providing a controlled, protective, and bioactive environment for microbial delivery. First, hydrogels can protect the viability of *P. histicola* during gastrointestinal transit by shielding the bacteria from gastric acidity and premature enzymatic degradation. Their ability to release encapsulated content in a controlled manner—adjustable via pH-sensitive or enzyme-degradable crosslinkers—ensures sustained colonization in the lower gastrointestinal tract, where immune modulation is most effective. This mimics the repeated oral administration protocols used in HLA-DQ8 murine models, thereby improving the reproducibility of microbiome-based immunotherapy studies. Second, the mucoadhesive and porous nature of hydrogels can be tailored to favor microbial adhesion to intestinal epithelium and to simulate physiological oxygen and nutrient gradients, facilitating the colonization of *P. histicola* and enhancing its local immunoregulatory effects. These include the suppression of Th17 responses and a concomitant rise in Tregs, both of which are associated with clinical improvement in RA.

From a translational perspective, oral hydrogel formulations—designed as capsules or mucoadhesive matrices—can serve as clinically feasible delivery systems for *P. histicola*, paving the way for future human trials. Finally, hydrogel-based delivery enhances the resolution of proteomic and immunologic studies by enabling precise temporal sampling of intestinal and systemic biomarkers. This facilitates the identification of microbial signatures linked to disease modulation, including those observed in RA patients, where reductions in *P. histicola*, *Bacteroides plebeius*, *Bifidobacterium bifidum*, and *Lactobacillus casei* have been documented.

### 7.2. Hydrogel with Polylysine Against S. aureus in Infected Wound Models

Lysine is an essential amino acid for humans, available in two enantiomeric forms: L-lysine and D-lysine. It can be synthesized through condensation polymerization or fermentation processes [115]. Polylysine is considered an ideal polymer for the development of antimicrobial hydrogels due to its potent antibacterial properties against both Gram-positive and Gram-negative bacteria, such as *S. aureus* and *Escherichia coli.* However, its high water solubility presents a significant limitation, as it may compromise the structural stability and sustained performance of hydrogel-based systems [116,117]. In vivo studies in C57BL/6 mouse models have reported low levels of inflammation following two weeks of exposure to zwitterionic polypeptide hydrogels containing alternating lysine sequences. These materials demonstrated reduced expression of inflammatory markers, including TNF-α, IL-6, and CCR7. Furthermore, fibrosis analysis at 3 and 6 months revealed limited extracellular matrix deposition, as assessed by Masson’s trichrome staining. Notably, these hydrogels minimized foreign body responses, highlighting their potential for biomedical applications in tissue engineering [118].

Among the components used in combination with hydrogels, polylysine exhibits potent antibacterial activity, effectively eliminating Staphylococcus aureus and Escherichia coli. Glycopeptide-based hydrogels composed of oxidized dextran (OD), polylysine (PL), dopamine (DA), and basic fibroblast growth factor (bFGF) demonstrate excellent structural integrity, injectability, adhesive properties, swelling capacity, and biodegradability. These hydrogels also exhibit pH-responsiveness due to the presence of Schiff base linkages, allowing them to respond to the acidic microenvironment of bacterially infected wounds and release their encapsulated agents accordingly. Dopamine efficiently scavenges reactive oxygen species (ROS) and promotes the polarization of macrophages toward the M2 phenotype, thereby mitigating oxidative stress. Meanwhile, bFGF upregulates CD31 and vascular endothelial growth factor (VEGF) expression, enhancing angiogenesis. These combined functionalities validate polylysine-containing hydrogels as promising candidates for accelerating wound healing in S. aureus-infected diabetic mouse models [119].

Hydrogels have also been explored for the repair and treatment of gastro-intestinal disorders. Procedures such as gastrotomy can benefit from hydrogel-based interventions. Recent studies have reported that alginate and hyaluronic acid-based hydrogels loaded with polylysine and manganese dioxide nanoparticles effectively reduce bacterial colonies of Staphylococcus aureus and Escherichia coli, with the antibacterial efficacy correlating with the concentration of polylysine. In vivo gastrotomy models in rabbits demonstrated enhanced tissue regeneration, as evidenced by increased fibroblast presence and gland formation after 7 days of treatment compared to controls, as observed in H&E-stained tissue sections. Additionally, Masson’s trichrome staining revealed significantly higher collagen deposition (46.9%) in the hydrogel-treated group compared to the control (33.2%). Furthermore, this hydrogel formulation significantly reduced the expression of pro-inflammatory cytokines, including TNF-α, IL-6, and IL-1β, supporting its anti-inflammatory and angiogenic potential for gastro-intestinal tissue repair [120].

## 8. Current Challenges

Hydrogels, whether synthetic or natural, have characteristics that make them suitable for biomedical applications. Their chemical and physical properties can be manipulated, enabling customized designs tailored to specific applications. Industrial manufacturing requires adherence to legal compliance and good manufacturing practices to ensure the quality, stability, and safety of functionalized biomaterials.

### 8.1. Poor Mechanical Properties

Hydrogels derived from natural polymers such as alginate, chitin, collagen, or chitosan exhibit significantly lower mechanical strength and stiffness compared to their synthetic counterparts, which limits their applicability in load-bearing environments such as tissue engineering scaffolds or wound dressings exposed to mechanical stress or movement. This mechanical fragility is largely attributed to their high water affinity, soft consistency, and loosely crosslinked polymer networks. These materials typically present a markedly lower Young’s modulus and reduced compressive strength relative to synthetic hydrogels, which often precludes their use in anatomically demanding sites such as bone or cartilage [121]. For instance, pure gelatin-based hydrogels may exhibit a Young’s modulus of approximately 48 kPa, a value insufficient for supporting tissues that require higher mechanical rigidity [122]. Moreover, their viscoelastic and poroelastic behavior, combined with a limited density of crosslinking points, renders them susceptible to mechanical trauma and fatigue under repetitive loading conditions [123].

### 8.2. Scalability and Production Costs of Hydrogels

Natural hydrogels, including those derived from polymers such as alginate, chitosan, hyaluronic acid, and collagen, have garnered significant attention in bone tissue engineering due to their biocompatibility, biodegradability, and resemblance to the extracellular matrix. However, their large-scale application faces critical challenges, particularly regarding manufacturing scalability and cost-effectiveness.

One of the most pressing limitations is the batch-to-batch variability inherent to natural polymers. Variations in molecular weight, purity, and degree of acetylation/deacetylation lead to inconsistent mechanical and biological properties, demanding rigorous quality control systems that significantly elevate production costs and compromise reproducibility at industrial scale [124].

Moreover, hydrogels possess a high water content, rendering them susceptible to dehydration, microbial contamination, or physicochemical degradation during storage or transportation. Preservation strategies such as lyophilization or cryopreservation require additional processing steps, which considerably increase production costs [125].

### 8.3. Interindividual Microbiome Variation

Numerous studies have demonstrated that the composition of the human microbiome exhibits marked interindividual variability across multiple anatomical sites, including the gastrointestinal tract, skin, oral cavity, and vaginal mucosa. This heterogeneity is largely influenced by intrinsic factors such as age, host genetics, dietary habits, and geographical origin, and should be carefully considered in the design of microbiota-targeted therapeutic strategies, including hydrogel-based delivery systems [126].

In particular, gut microbiota analyses have revealed a distinct distribution pattern of dominant bacterial genera across populations. For instance, individuals residing in Western countries—primarily of Caucasian descent—tend to harbor Bacteroides-dominant communities, whereas Prevotella predominates in individuals from non-Western regions, such as African and Asian populations. This divergence has been attributed mainly to dietary patterns, with Western diets typically high in fat and low in fiber, in contrast to the high-fiber, low-fat diets common in non-Western populations [127].

Furthermore, longitudinal investigations of the skin microbiome indicate that interindividual variation represents the primary determinant of bacterial community composition, outweighing anatomical location or disease status. Each person maintains a unique and relatively stable microbial fingerprint over time and across various skin layers [128].

Emerging genomic evidence also supports the role of host genetic factors in shaping the wound microbiome. A genome-wide association study in individuals with chronic wounds identified a significant correlation between host genetic background and the relative abundance of specific bacterial taxa, highlighting the influence of host genetics on microbial community structure and wound healing dynamics [126]. In addition, metagenomic studies have shown that strains of the same microbial species may differ by approximately 13% in gene content between individuals, thereby influencing metabolic function and potentially altering the efficacy of probiotic-loaded hydrogels in personalized therapeutic applications [129].

### 8.4. Reproducibility in the Fabrication of Multi-Functional Hydrogels

The functionalization of biomaterials, such as hydrogels, for biomedical and pharmaceutical purposes is an area of increasing interest. Hydrogels have been explored as biocompatible biomaterials in applications ranging from simple matrices for the release of active compounds or maintaining local hydration to sophisticated, multi-functional systems. However, the design and small-scale manufacturing of hydrogels present significant challenges during experimental or preclinical stages. While sophisticated technologies are used to manufacture multifunctionalized hydrogels with biological components, these technologies limit their accessibility in the global market. Furthermore, the multistage processes required to synthesize these biomaterials at the industrial level increase unit operations and production costs. Additionally, the manual steps required for designing multifunctionalized matrices in experimental hydrogels can result in high batch variability and low yields. Therefore, the feasibility of production, reproduction, handling, and storage, as well as regulatory aspects, must be evaluated to ensure their widespread use [130].

### 8.5. Risk of Immunogenicity in Hydrogels with Bacterial Components

The polymers used to create hydrogels, whether natural, synthetic, or a mixture of both, must exhibit biocompatibility with the body’s cells, tissues, and fluids. Furthermore, the materials must be biodegradable, nontoxic, and not trigger chronic immune and inflammatory reactions [35,131]. There is evidence of the beneficial effects of modifying the skin microbiome in conditions of dysbiosis. However, it is necessary to study the harmful effects in depth that its alteration can generate. In the case of hydrogels functionalized with microorganisms or their components and metabolites, evaluating biocompatibility becomes relevant. One of the strategies to restore the dysbiosis caused by a wound (mainly chronic ones) in the resident microbiome consists of the use of prebiotic compounds. This is intended to promote the growth of microorganisms that protect the skin barrier, which, in turn, are essential in modulating physiological responses to the detection of pathogens related to infectious processes in wounds. In turn, the use of microorganisms such as *Lactobacillus plantarum* as a protective agent against infections caused by Pseudomonas spp. in chronic wounds, including burns, has been explored as a probiotic treatment [6,7]. Furthermore, the inclusion of postbiotics from microorganisms considered beneficial for immune and cellular modulation has begun to be explored in the context of using postbiotic strains. Golkar et al. (2021) evaluated the healing effect of strains of *Lactobacillus fermentum*, *Lactobacillus reuteri*, *and Bacillus subtilis* sp. natto for the treatment of acute skin wounds in murine models [132]. However, this type of therapeutic strategy can have an adverse effect, generating immune exacerbation and promoting inflammatory processes that hinder wound healing and generate greater dysbiosis in the resident microbiome. Yanamandra et al. report an example of this effect. (2022), using two modified *E. coli* strains encapsulated in a Pluronic F127 diacrylate hydrogel as living therapeutic material (LTM). The objective of this research was to evaluate the immunogenicity of the bacterial hydrogel and its effect on stimulating pro-inflammatory cytokines and cytotoxic proteins in human peripheral blood mononuclear cells (PBMCs), as well as the subsequent activation of T cells and natural killer cells. The results showed a lower immunological effect in the bacterial hydrogel with the endotoxin-free strain, which prevented apoptosis of the immune cells and triggered the release of IL-6 from the surviving PBMC cells [91]. Therefore, for the use of bacterial LTMs in hydrogels for biomedical applications, it is essential to measure the immune responses they may elicit. Their immunogenicity can be correlated with the scaffold material and the living bacterial components present in the polymeric matrix. Although bacteria are not in direct contact with the various cells of the body, the immune response they trigger could be associated with the detection of metabolites, secreted molecules, dead bacteria, and components related to their degradation.

## 9. Future Perspectives and Emerging Trends

### 9.1. Predictive Modeling to Design Hydrogels Adapted to the Patient’s Microbial Profile

Among the many applications of hydrogels, several require interaction with the patient’s microbial profile [133]. For example, hydrogels loaded with antibacterial molecules have been developed and tested with good results [134]. However, there is always interest in perfecting the techniques and models. Therefore, the next step in this area would be to utilize emerging technologies, such as artificial intelligence, computational modeling, and omics analysis, to design hydrogels that are fully adapted to the patient’s microbial profile. This would achieve the goal of having 100% customizable therapies designed explicitly for the microbial environment at the application site. This would allow for more effective antibiotic therapies, prevent antibiotic resistance (due to the use of inappropriate antibiotics), and reduce unnecessary antibiotic exposure for the patient.

The advantages of having hydrogels tailored to the patient’s microbial profile are numerous and considerable. By analyzing the patient’s microbial profile before designing the hydrogel, the base chemical compound (chitosan, alginate, cellulose, hyaluronic acid, and others) to be used can be selected [134]. The type of antibacterial molecules it will carry and the release mechanism can also be specified [133]. All of this seeks more effective therapies, with the correct drug, dosed and released appropriately, to optimize the bactericidal and antifungal processes. Microbial removal is a crucial step for hydrogels for therapeutic purposes to function correctly. By eliminating microbial agents, inflammatory processes are controlled, allowing for the repair and regeneration of tissues.

### 9.2. CRISPR-Activated Hydrogels to Edit Pathogen or Commensal Bacterial Genes In Situ

Advanced hydrogels use DNA to control how they react to specific things. This makes them useful for detecting biological substances, delivering medicine, and treating illnesses. A DNA hydrogel is a water-loving polymer that uses DNA as a main part of its structure or to connect other synthetic polymers [135,136]. Some researchers have added CRISPR-Cas to DNA hydrogels to make them more responsive and easier to customize. CRISPR enzymes can change the properties of hydrogels using DNA parts, including a “switcher” and an “actuator.” The switcher has a Cas12a-gRNA complex and a target DNA strand. Its open and closed states are controlled by how well the gRNA and target DNA bind together [137,138]. The actuator contains cleavable ssDNA cross-linkers that actively tune the hydrogel’s properties. Integrating the CRISPR-Cas toolbox with hydrogels enables controlled release of small molecules, nanoparticles, and live cells in response to a specific target. This CRISPR-associated hydrogel system has potential applications as a biosensor for detecting viral pathogens and distinguishing between pathogenic and non-pathogenic bacteria [135].

Another approach is to use CRISPR to create hydrogels that change their form on command [139]. These shape-shifting materials can be used to deliver small molecules and sense biological signals. Collins and his team worked with DNA hydrogels combined with CRISPR-Cas12a because the Cas12 enzyme can be programmed to recognize a specific DNA sequence, which breaks up after Cas12a recognizes the target sequence in response to a stimulus [139]. The breakup of the single DNA strands triggers the hydrogels to change shape or, in some cases, completely dissolve, releasing a payload. A hydrogel has been created that is programmed to release enzymes, small molecules, and even human cells in response to stimuli. Some investigators hope that these hydrogels could be used to make smart therapeutics that release, for example, cancer drugs in the presence of a tumor, or antibiotics around an infection.

Soon, CRISPR-activated hydrogels will be designed to edit the genes of pathogenic or commensal bacteria in situ. This will revolutionize the applications of hydrogels, potentially even reducing or eliminating the need to load them with antibiotics to combat infections, or modulating the multiple bacterial flora found in the human body.

### 9.3. Use of Marine-Derived Polypeptides or Recombinant Collagen to Reduce Costs and Risks

A key area of opportunity in the design and development of hydrogels is the pursuit of reducing costs and mitigating potential risks for end-users. This is intended to develop a therapy approved for use in humans. Two options that could be explored soon are the use of marine polypeptides or recombinant collagen to design hydrogels. Collagen is the major component of bones, skin, cartilage, and tendons [140]. Marine collagen is considered safer and easier to extract compared to mammalian collagen because it poses fewer risks of transmitting viral diseases and has been proven to be less immunogenic, potentially avoiding secondary complications when used in the human body [141]. Also, marine collagen contains high amounts of hydroxyproline, an amino acid essential for skin, blood vessels, and other connective tissues, which can be used in tissue engineering [142]. On the other hand, recombinant collagen is a type of collagen produced through genetic engineering rather than being extracted from animal or human tissues. It is synthesized by inserting collagen genes into host organisms (such as bacteria, yeast, or mammalian cells), which then produce collagen proteins in a controlled laboratory environment. Among their characteristics, they are made without animal-derived biomolecules. They are free of contaminants, including viruses, prions, and immunogenic components. They can be designed to produce a modified amino acid sequence that improves stability, solubility, or specific biological functions. They also reduce the risk of allergies or disease transmission.

In the future, marine-derived collagen and recombinant collagen are expected to be utilized in the development of hydrogels for therapeutic purposes, due to their lower cost and reduced risk of an immunogenic response from the final user. This enables the widespread adoption of hydrogels in the biomedical field.

## 10. Conclusions

The reviewed evidence highlights the transformative potential of hydrogels as multi-functional biomaterials for modulating the microbiome in tissue regeneration and infection control. Through the encapsulation and controlled delivery of probiotics, antimicrobial agents, and bioactive molecules, hydrogels demonstrate the capacity to restore microbial homeostasis, promote epithelial repair, and modulate innate and adaptive immune responses. The successful application of hydrogels in protecting probiotics, such as *Lactobacillus* spp. and *Prevotella histicola*, from environmental stressors, to enhance their therapeutic efficacy in models of inflammatory diseases, including rheumatoid arthritis and colitis, is a new field to explore.

Additionally, hydrogel systems functionalized with nitric oxide donors, silver nanoparticles, and postbiotic components exhibit potent antibiofilm and antimicrobial properties, particularly against pathogens like Staphylococcus aureus and *Pseudomonas aeruginosa*. Innovative technologies such as 3D bioprinting, photothermal therapy, and self-healing materials further expand the applicability of hydrogels to complex wound environments and irregular tissue defects. However, challenges persist regarding immunogenic risks, reproducibility, and regulatory standardization for clinical translation. Future perspectives point toward the integration of omics technologies, computational modeling, and patient-specific microbial profiling to design precision hydrogels that align with individual microbiome signatures. Overall, the convergence of hydrogel engineering and microbiome science opens a promising avenue for next-generation regenerative therapies and personalized medicine.

## Figures and Tables

**Table 1:** Overview of biomaterials and crosslinking methods for probiotic/prebiotic delivery.

Biomaterial/Crosslinking	Probiotics/Prebiotics	Reference
Alginate microbeads/Ionic crosslinking	*Lactobacillus plantarum* or *Lactobacillus rhamnosus*	[38]
Hyaluronic acid hydrogel/Photo-crosslinking	*Lactobacillus paracasei*/EPS-M76	[40]
Alginate microbeads/Ionic crosslinking	*Lactobacillus acidophilus*/Reishi mushroom extract	[41]
Alginate Microbeads/Ionic crosslinking	*Enterococcus. faecium* or *Aerococcus viridans*/Inulin or apple marc flour	[43]
Fructooligosaccharides-Alginate microbeads/Ionic crosslinking	*Lactobacillus rhamnosus*/Fructooligosaccharides	[44]
Alginate microbeads/Ionic crosslinking	*Lactobacillus rhamnosus*/provitamin A	[55]
Chitosan-coated alginate microbeads/Ionic crosslinking	*Lactobacillus gasseri* or *Bifidobacterium bifidum*/Quercetin	[46]
Gelatin hydrogel/Enzymatic crosslinking	*L. plantarum*	[47]
Alginate-gelatin microbeads/Ionic crosslinking	*Lactobacillus plantarum*	[48]
Alginate microbeads/Ionic crosslinking	*Bacillus coagulans*	[56]
Bovine serum albumin hydrogel/Chemical crosslinking	*E. coli*	[57]
Heparin-poloxamer hydrogel/Thermosensitive crosslinking	*L. lactis*	[58]
Gelatin microbeads/Photo-crosslinking	*Cutibacterium acnes*-derived extracellular vesicles	[59]
PF-127 hydrogel/Thermosensitive crosslinking	*Parabacteroides goldsteinii*-derived outer membrane vesicles	[60]
Poly (vinyl alcohol)-Gelatin/Ionic crosslinking	*Lactiplantibacillus plantarum*	[61]
Methacrylate-Hyaluronic acid/Ionic crosslinking	*Lactobacillus reuteri*	[62]
Polyethylene glycol-alginate/Ionic crosslinking	*Lactobacillus acidophilus*	[63]

**Table 2:** Biomaterial-based systems for antibacterial and antibiofilm formation.

Biomaterial/Hydrogel System	Microorganism(s) Affected: Antibacterial and Anti-Biofilm Formation	Reference
PSPG hydrogel with Pt-decorated AuNPs + SNP-loaded PCN	General spectrum	[65]
Quaternized chitosan hydrogel + BNN6 in MPDA + NIR activation	*Staphylococcus aureus*	[66]
Chitosan hydrogel + PG@Arg/IR820	*S. aureus*	[67]
S-nitrosothiolated gelatin hydrogel	*Escherichia coli*, *Staphylococcus aureus*	[68]
Chitosan-stabilized AgNPs gelatin hydrogel	*S. aureus*, *B. subtilis*, *P. aeruginosa*, *E. coli*	[70]
AgNPs and AuNPs in chitosan matrix	*S. aureus*, *P. aeruginosa*	[71]
LTF-functionalized AgNPs in gelatin hydrogel	*S. aureus*, *P. aeruginosa*	[72]
AgNP + LTF + DsiRNA in gelatin hydrogel	*S. aureus*, *P. aeruginosa*	[73]
Poly (ethylene glycol) DFO-loaded hydrogel + MXene + NIR	Methicillin-resistant *S. aureus* (MRSA)	[74]
Alginate hydrogel + TA-AgNPs	*S. pyogenes*, *S. aureus*, *P. aeruginosa*	[75]
Low viscosity chitosan	*S. epidermidis*	[76]
Medium molecular weight native chitosan	*Listeria monocytogenes*, *B. cereus*, *S. enterica*, *P. fluorescens*	[77]
Chitosan-coated catheters	*S. epidermidis*, *C. albicans*	[78,79]
Carboxymethyl-chitosan hydrogel	*S. epidermidis*, *C. tropicalis*	[80]
Chitosan hydrogel with epsilon-poly-L-lysine	*P. aeruginosa*, *S. aureus*, *C. albicans*	[81]
Chitosan-coated surfaces	*K. pneumoniae*, *P. aeruginosa*, *C. albicans*	[82]
Chitooligosaccharides (COS)	*S. aureus*	[83]

**Table 3:** Biomaterial-based systems for immune modulation: in vivo evidence summary.

Biomaterial/Hydrogel System	Application In Vivo Study/Target Microorganism	Key Molecules Involved	Cytokines Regulated	Reference
Calcium alginate hydrogel + 5-aminosalicylic acid	Colitis model/Gut microbiota	5-aminosalicylic acid	↓ IL-6, IL-1β, TNF-α; ↑ IL-10	[94]
Heparin–poloxamer hydrogel + *Lactococcus lactis*	Diabetic wound model inflammation/skin microbiota modulation	Lactic acid and VEGF	↓ TNF-α, iNOS, NO; ↑ IL-10, CD206, ARG1	[58]
Oxidized konjac glucomannan-chitosan-arginine hydrogel + protocatechualdehyde, Fe^3+^	Full-thickness MRSA-infected wounds/skin microbiota modulation	Protocatechualdehyde, Fe^3+^	↓ TNF-α; ↑ IL-10, CD206	[95]
Gelatin methacrylate hydrogel + *Cutibacterium acnes*-derived extracellular vesicles	Psoriasis model/skin microbiota modulation	Microbial extracellular vesicles	↓ IL-17, IL-22; ↑ IL-10	[59]
PF-127 hydrogel + *Parabacteroides goldsteinii*-derived outer membrane vesicles	Psoriasis model/skin microbiota modulation	Outer membrane vesicles (pentadecanoic acid)	↓ IL-23, IL-17; ↑ IL-10	[60]
Hyaluronic acid-*Bacillus velezensis* extracellular polysaccharides + *Lactobacillus paracasei*	Wound healing model/skin microbiota modulation	Lactic acid	↓ TNF-α; ↑ IL-10, VEGF-α	[40]
Hyaluronic acid-gelatin hydrogel + jileicin	MRSA-infected diabetic wounds model/ skin microbiota modulation	Jileicin (bacteriocin)	↑ IL-10; ↓ TNF-α	[97]
Poly (ethylene glycol) diacrylate gelated peritoneal macrophages + *E. coli* Nissle	Intestinal inflammation/Gut microbiota	TNFR2, IL1R2, TLR4, *E. coli* Nissle 1917	Neutralization of TNF-α, IL-1β	[90]

## Data Availability

Not applicable.
